# HPVbase – a knowledgebase of viral integrations, methylation patterns and microRNAs aberrant expression: As potential biomarkers for Human papillomaviruses mediated carcinomas

**DOI:** 10.1038/srep12522

**Published:** 2015-07-24

**Authors:** Amit Kumar Gupta, Manoj Kumar

**Affiliations:** 1 Bioinformatics Centre, Institute of Microbial Technology, Council of Scientific and Industrial Research (CSIR), Sector 39-A, Chandigarh; 160036, India

## Abstract

Human papillomaviruses (HPVs) are extremely associated with different carcinomas. Despite consequential accomplishments, there is still need to establish more promising biomarkers to discriminate cancerous progressions. Therefore, we have developed HPVbase (http://crdd.osdd.net/servers/hpvbase/), a comprehensive resource for three major efficacious cancer biomarkers i.e. integration and breakpoint events, HPVs methylation patterns and HPV mediated aberrant expression of distinct host microRNAs (miRNAs). It includes clinically important 1257 integrants and integration sites from different HPV types i.e. 16, 18, 31, 33 and 45 associated with distinct histological conditions. An inclusive HPV integrant and breakpoints browser was designed to provide easy browsing and straightforward analysis. Our study also provides 719 major quantitative HPV DNA methylation observations distributed in 5 distinct HPV genotypes from higher to lower in numbers namely HPV 16 (495), HPV 18 (113), HPV45 (66), HPV 31 (34) and HPV 33 (11). Additionally, we have curated and compiled clinically significant aberrant expression profile of 341 miRNAs including their target genes in distinct carcinomas, which can be utilized for miRNA therapeutics. A user-friendly web interface has been developed for easy data retrieval and analysis. We foresee that HPVbase an integrated and multi-comparative platform would facilitate reliable cancer diagnostics and prognosis.

The human papillomaviruses (HPVs) are circular, double-stranded DNA genome of approximately 8.0 kb in length. It belongs to the papillomaviridae family, which is further taxonomically classified into distinct genera namely alpha, beta, gamma, mupa and nupa^1^. HPVs encode eight well-defined open reading frames (ORFs) along with one non-coding long control region (LCR) or regulatory region. HPV proteins are mainly divided into two coding regions classified as early (E) and late (L) region. E region includes six ORFs encoding 3 functional regulatory genes (E1, E2, E4), 3 oncogenes (E5, E6, E7) and the L region encodes the two viral capsid genes (L1 and L2)^1^,^2^,^3^.

HPVs are well adapted in host differentiating epithelial tissues and prevalent in cervical carcinogenesis. They employ cellular machinery to survive in host. Expression and functions of viral proteins primarily HPV oncoproteins E6 and E7 is very essential for the tumor progression. Upregulation of these oncoproteins in the infected cells have been shown to expedite proliferation, immortalization and malignant transformation. They interact with numerous cellular targets mainly inhibits two essential tumor suppressor proteins, p53 and retinoblastoma (Rb). Interaction of E6 with p53 prohibits proteolytic degradation and abrogates apoptosis^4^. In addition, binding of E7 to pRb inhibits cell cycle arrest^5^,^6^.

Cervical carcinoma (CC) is the fourth most common cancer and cause of death in women worldwide (GLOBOCAN 2012). Series of steps involves in cervical oncogenesis are HPVs infection and transmission, viral persistence, progression of infected epithelium to precancer or high-grade precursor lesion and invasion. Persistence infection with distinct carcinogenic HPVs is a critical factor in the etiology of cervical precursor lesions and cancer^2^,^7^,^8^. Prodigious involvement and influence of HPVs in distinct carcinomas has accelerated the research in screening and prevention strategies for cancers. Moreover, cytology and HPV-based screening methods have greatly reduced the cancer morbidity and mortality rate among women^9^. However, despite of consequential accomplishments, sensitivity and specificity of these methods remains a challenge to make decision for colposcopic referral of women. Furthermore, these are not sufficient to discriminate transient infection with high-grade carcinomas^10^. Additionally, there is no effective treatment and therapeutics for HPV infection is available to eradicate the CC up to now and it still remains prevailing cause of cancer death^8^,^10^.

HPV infection and associated consequences are valuable determinants of multistage cervical neoplastic progression. In this context, there are alternative biomarkers, which could discriminate latent infection with high-grade precursor lesion and eventually cancer^8^. Potential biomarkers pertinent to HPV mediated diseases are mainly viral DNA integration, viral methylation and cellular miRNAs expression. These could improve the reliability of cancer screening and prevention^8^,^11^.

The infection and integration of HPVs is a well-known factor and event in the development of CC, human genital and head and neck squamous cell carcinoma (HNSCC)^12^,^13^,^14^,^15^,^16^,^17^,^18^. They are known to be integrating into human chromosomes during the progression of cancers, which provide enhancement and stabilization of HPV oncogenes (E6 and E7) transcription^19^,^20^. These integration sites found to be distributed all over the human genome with a preference in or near to fragile sites (FS) or oncogenes and translocation breakpoints^21^. It usually rises in high-grade lesions and carcinomas with uneven integration frequency in distinct cases^12^,^22^. However, in case of low-grade lesions and benign, HPV genomes persist mainly in episomal state^23^.

In addition, epigenetic mechanisms are also known to influence chromatin conformation and alter gene expression. CpG methylation is a fundamental and well-recognized molecular regulatory mechanism in epigenetics^24^,^25^. Methylations play a crucial role in the HPV transcriptional modulation. Although the underline molecular basis and determinant of methylation at individual CpG sites during the transformation is still poorly understood^26^. The major observations from previous studies, guide towards two distinct types of molecular mechanisms. One, targeted methylation may contribute in neoplastic progression by blocking binding sites (E2BSs) of HPV E2 repressor, which stimulate oncogene (E6 and E7) expression, thus enhance the carcinogenesis. Second, it may be a consequence of host defense mechanism against integrated HPV genome for the inhibition of viral replication and transcription through de novo methylation^27^,^28^,^29^.

Apart from the general concepts of genomic instability govern by carcinogenic HPVs the mechanism of transformation is more complex. The viral oncoproteins (E6 & E7) are known to interact with several cellular components and may affect different cellular pathways^6^. The viral oncogenes E6 and E7 deregulate tumor suppressive and oncogenic miRNAs (Oncomirs)^30^,^31^. In addition, host miRNAs also influence the papillomavirus genes expression by targeting viral RNA transcripts thus provide an interplay mechanism between cellular miRNAs and HPV oncogenes^32^. Mature miRNAs are approximately 18–25 nucleotides in length and are found in the wide range of organisms including viruses^33^. MiRNAs alters the gene expression either by repressing mRNA translation or by catalyzing the mRNA cleavage^34^,^35^. They usually affect various molecular processes such as apoptosis, cell proliferation, morphogenesis, human tumorogenesis, chromatin modifications etc^36^.

Despite of numerous experimental studies in the field, there is a paucity of computational resources with a unique focus on cancer progression and therapeutically potential biomarkers. Moreover, it is still unclear and less known about the viral mediated processes, such as integration events, methylation pattern in different epidemiological conditions and above all importance of various cellular miRNAs expressions in the etiology of oncogenesis. Hence, it is most important and necessary to have a proper multi-comparative platform of these specific biomarkers. Here, the goal of “**HPVbase**” is to provide a comprehensive web-based resource of the extensively cross-analyzed different biomarkers.

## Results

### HPV integration and breakpoints

The importance and crucial role of HPV integration and human genome integration sites in the progression and etiology of CC were identified and analyzed in several studies. However, complete knowledge and understanding of integration mechanism is still unclear. An inclusive HPV integrant and breakpoints browser were designed to provide analysis and interpretation. In total, 1257 integrants and integration sites were collected which are associated with distinct histological conditions. These integration sites are distributed among several HPV types mainly on HPV16, 18, 45, 33 and 31. It comprises of various clinical information such as viral integrant, host integration sites, gene target region, fragile sites, cancer specimen type, sample size, carcinoma types, detection techniques etc.

Using the highly interactive browser user can find and browse distinct HPV integrants. Different color-coded blocks provide corresponding information and existing analysis of HPV integration (see Supplementary Figure S1). All clinical histological information was comprehended using the set of tracks. Users can easily find respective details regarding integration events and utilize it to map and compare with new experimental data. Supplementary Figure S2 shows the distribution of integration sites among distinct HPV types. Most of these sites belong to HPV16 (954), HPV18 (216), HPV45 (33) and HPV33 (33). Additionally, we have also reported and depicted frequency distribution of HPV integration events based on specific disease (cancer) types and chromosomal loci (see Supplementary Figure S3-S4). As shown in Supplementary Figure S5, integration data in HPVbase is also represented in user-friendly tabular format with supporting additional annotations with reference links. Users can find exact chromosomal location, cytoband and target host genes using the HPV integration and breakpoints table. This knowledge can be utilized to optimize and accelerate clinical biomarker research.

**Figure 1:**
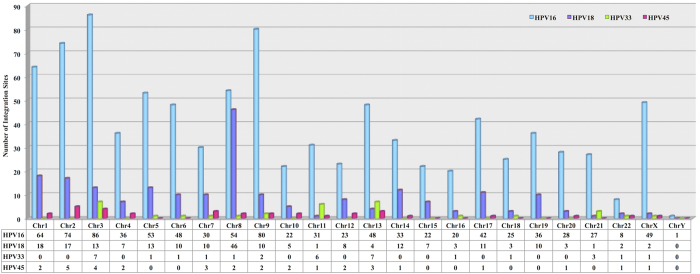
Bar chart representing distribution of HPV16, HPV18, HPV33 and HPV45 integration sites on human genome.

The most important and critical part of integration events is their frequency to integrate on different chromosomes and genomic region. To facilitate this, we have also analyzed HPV type wise distribution and frequency of integration and breakpoints on different human chromosomes. Figure 1 depicts chromosomal distribution frequency of HPV16, 18, 33 and 45 integration sites respectively. Using this we can find prominent genomic target regions in human genome liable for higher instability. As delineated in Fig. 1, HPVs integration sites and breakpoints are distributed all over the human genome but some genomic regions show higher liability towards disruption and can be used as the hot spot for cancer clinical research. We found that HPV16 is used to prefer and highly integrate at chromosome 3, 9, 2, 1, 8 and 5. Similarly, HPV18 mainly favors chromosome 8, 1, 2, 5 and 3. Likewise, HPV33 and HPV45 integration sites are almost evenly distributed among different locations.

**Figure 2:**
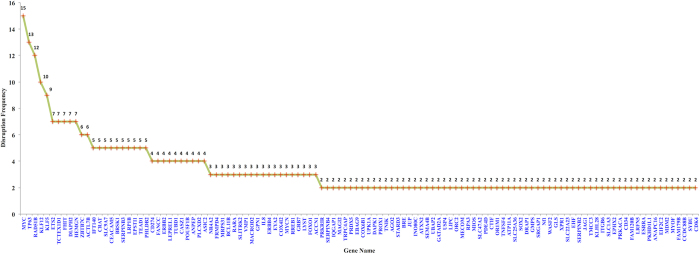
Depicting frequency of genes disrupted due to viral integration events.

Moreover, we have also analyzed the complete gene compendium altered/disrupted due to integration events presented in HPVbase. First of all, we have extracted missing gene name information based on human genomic coordinates using UCSC genome browser. Subsequently, from entire set after removing duplicates and other non-genes (pseudo genes, LINCs, non-coding RNAs) we have listed 481 unique genes. Furthermore, disruption frequencies of each unique 481 genes from whole dataset were calculated (see additional file 2 Table S3). Figure 2 portrays frequencies of disrupted genes, which are at least represented twice in complete gene set. These genes are predominantly involved in metabolic, biological regulations and apoptotic processes.

### HPV methylation patterns

Extensive research in the quantitation of methylation in the carcinogenesis allowed searching methylation patterns in different diseased stages. Our study provides major quantitative HPV DNA methylation observations together and the probable combination of possible CpG methylation sites that could be used to discriminate normal and cancerous progressions. Distinct studies provide different notations for methylation status. To simplify the terminologies, based on methylation levels we have used three categories: hypo or low methylation (HypoM), hyper or high methylation (HyperM) and significantly hyper methylation (HyperM##). Here, we have cataloged 719 methylation entries along with respective important and clinical information. It comprises of CpG methylation sites, gene region, differential methylation patterns, detection techniques, sample size and disease sample type use to identify methylation status (see Supplementary Figure S6). These are distributed in 5 distinct HPV genotypes from higher to lower in numbers namely HPV16 (495), HPV18 (113), HPV45 (66), HPV31 (34) and HPV33 (11) (see Supplementary Figure S7).

Assorted cancer studies have advocated an association between CpG methylation patterns and the carcinogenic development. Integration of episomal HPV genomes in carcinoma usually correlates with elevated DNA methylation. Our resource also provide diverse pattern in methylation outcome in corresponding specimen type, histological conditions and detection techniques used. In HPV positive women, the methylation level at specific CpGs increase with consistent viral infection and even enhance more remarkably in high-grade lesions. Maximum sites from late HPV regions L1 and L2 exhibit conclusive and significant hyper methylation patterns. However, methylation pattern in adjacent long control region (LCR) shows relative inconsistency. It could provide a comprehensive base for comparative examination between distinctive features and cancers, which in turn facilitate advancement in clinical screening tests. Furthermore, it could be used as a cost-effective molecular tool for the triage of HPV^+ve^ women.

### Cellular miRNAs aberrant expression

In various studies, miRNAs are suggested being involved in pathogenesis of cancers usually in CC and HNSCC including other cancer types i.e. breast cancer, thyroid papillary malignancy, ovarian cancer, prostate cancer etc^37^,^38^. The aberrant (increased or decreased) expression of miRNAs is evidentiary in diverse HPV mediated cancers and viral diseases^37^,^38^. Thus, the screening for differential expression profile of miRNAs in cancerous and normal tissues may help to examine the role of miRNAs in cancer pathogenesis.

To facilitate this, we developed an integrated source for the cellular miRNAs with description, which could be explored for greater insight between miRNAs and HPV related tumorogenesis. Regulation of cellular miRNAs is key to prognosis. We have curated and compiled clinically significant aberrant expression profile of 341 miRNAs from different carcinomas. It includes 142 miRNAs, which comprises of 80 upregulated and 62 downregulated in CC, 176 miRNAs among which 85 and 91 are up and downregulated respectively from HNSCCs and 22 miRNAs includes 9 upregulated and 13 downregulated from vulvar carcinoma. Beside this, 1 downregulated miRNA is related to penile carcinoma. It includes miRNAs and their mirbase id, host chromosomal location, genome coordinates, their potential target genes as shown in Supplementary Figure S8. These miRNAs affects various target genes, which mainly influence apoptosis, metastasis, cell proliferation, senescence, host defense mechanism, immune recognition and genomic stability with other host mechanisms. An expression profile of these miRNAs and their target genes can be utilized as the prognostic marker for aggressiveness of cancer.

**Figure 3:**
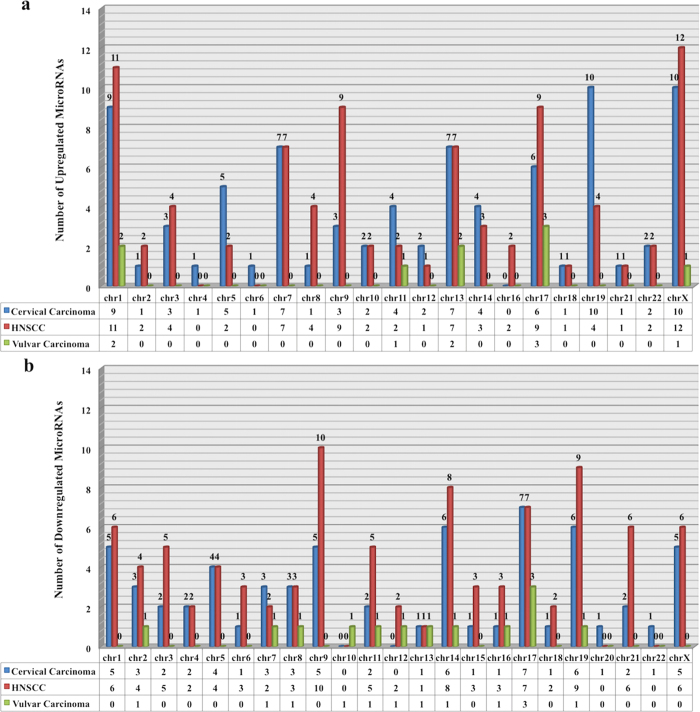
Plot showing chromosomal distribution of aberrantly expressed miRNAs in HPV associated carcinomas. (**a)** Upregulated miRNAs, (**b**) Downregulated miRNAs.

**Figure 4:**
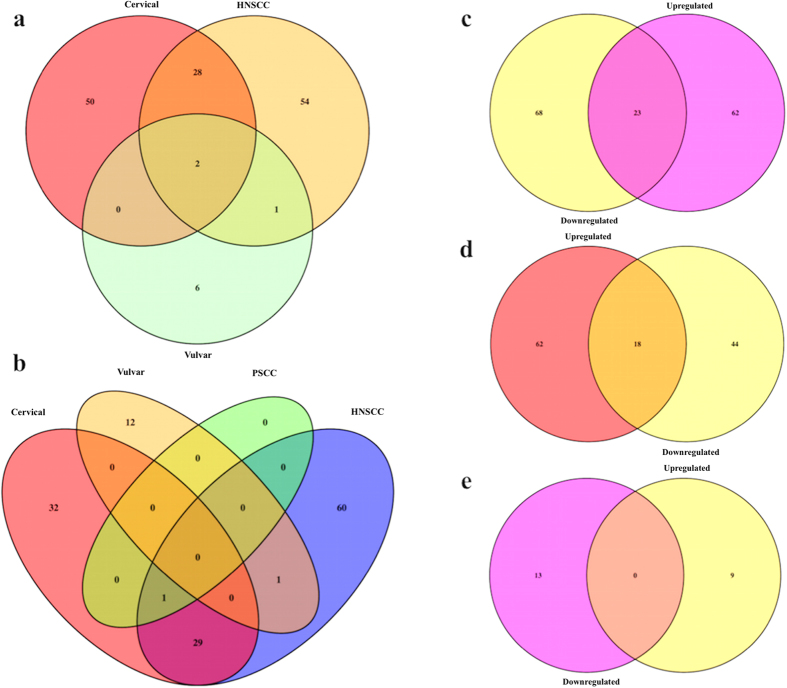
Figure illustrating commonly regulated miRNAs in diverse carcinomas. (**a**) Upregulated miRNAs, (**b**) Downregulated miRNAs, (**c**) Up and down regulated miRNAs in HNSCC, (**d**) Up and down regulated miRNAs in CC, and (**e**) Up and down regulated miRNAs in vulvar carcinoma.

We have cross-analyzed chromosomal distribution of these aberrantly (up and down) regulated miRNAs in different carcinomas i.e. CC, HNSCC and vulvar carcinoma. Figure 3 shows category wise distribution of these miRNAs on genomic region. Using this we can find chromosomal distribution relationship between integration hot spots and various miRNAs expression profile in distinct carcinoma. Along with this, we have also explored intra-relationship between these miRNAs expression profile and found set of commonly up regulated (see Fig. 4a) and down regulated (see Fig. 4b) miRNAs in diverse cancers. It is also interesting to notice some miRNAs shows both down and up regulation with in specific cancer type. We have identified 23 (see Fig. 4c) and 18 (see Fig. 4d) miRNAs, which are both over and under expressed in HNSCC and CC respectively. Dual expression can be a further area of research to find their specific role in varied conditions.

### Utility as potential biomarker

The vital role of HPV integrations and human genome chromosomal sites were analyzed and illustrated in various studies. HR-HPVs integration is a key incident in HPV-mediated carcinogenesis, which leads to increase expression and stability of viral oncogenes (E6/E7) as well as disruption of various key cellular proteins. It is usually considered as one of the main events takes place during the transition from low grade to high-grade lesions. Additionally, it was also observed that these are frequently allied with FS normally play a role in the structural alterations, translocations and large deletions. Thus, it is one of the important mechanisms that enhances and contributes in aberrant proliferation, genomic instability and cellular immortalization, which enhance malignant progression. It plays an acute function not only to alter viral genome as well as host genomic regions mainly associated with cancer related genes.

From our analysis, we have identify and reported certain specific chromosomal loci regions such as 8q24.21, 3q28, 13q22.1, 9q22.33, 14q24.1 and many more which shows higher level of viral integration indicating preferential target in diverse carcinomas (see Supplementary Figure S4). In this study, we have also catalogued and represented a comprehensive set of disrupted genes due to viral integration events (see Fig. 2). Among these genes most of them are interrelated with tumor development and various regulatory processes that could jointly become involved in viral induced oncogenesis. Such as RAD51B, TP63, MYC, ETS2, FHIT etc.

For example, TP63 is a tumor protein 63 belongs to family of transcription factors. It mainly involves in complex events and regulates cellular proliferation and neoplastic progression. MYC, it is a v-myc avian myelocytomatosis viral oncogene homolog usually plays a role in cellular transformation, apoptosis and cell cycle. ETS2, v-ets avian erythroblastosis virus E26 oncogene homolog 2 is a tumor suppressor gene mainly regulates genes involved in development and apoptosis. It also found to be involved in regulation of telomerase. FHIT, belongs to histidine triad protein family is a tumor suppressor gene. It encloses the common fragile site FRA3B and highly susceptible to the translocations and aberrant expression which leads to the various carcinomas. RAD51B (RAD51 paralog B) is a member of the RAD51 family. It is essential element of the DNA repair pathway and found to be associated with cell cycle delay and cell apoptosis. Hence, integration at unstable regions and at certain genes concomitant with DNA repair mechanism also signifies its relation with various chromosomal machineries.

These interpretations from existing knowledge suggest that prevalence of certain viral integration sites on specific genomic locations could play an essential role to determine eventual cancer phenotype. Therefore, revealing these imperative events in the transforming lesions from low grade to invasive carcinomas might be useful and precious to be used as potential cancer biomarker.

Apart, from genetic changes due to integration, HPVs are also known to cause epigenetic alterations mainly modification in DNA methylation pattern and miRNAs aberrant expression. These mechanisms contribute to the development of cancer either reducing expression of tumor suppressor genes or the enhancing oncogene expression supporting cancer progression. With the advent of high throughput technologies, researchers are now focusing on the application of these alterations to explore their prognostic and diagnostic value to determine disease progression^39^,^40^. The careful quest suggests that methylation patterns and rate differ according to pathological conditions and severities^41^,^42^. The profound research in quantification of HPV DNA methylation can be utilized as a predictive prominent biomarker to distinguish transient infection from those that evolve to cancerous state. Data from our resource could provide broad spectrum of HPV methylome from diverse cancers. This may help research community to identify certain combination of HPV methylation pattern to discriminate malignant from normal tissues.

Additionally, differential expression of oncogenic and tumor suppressive miRNAs has confounding affect on cancer paradigm. They generally post transcriptionally modulate or regulate expression of target genes and play significant role in cell cycle progression, apoptosis, angiogenesis etc. the observation of different circulatory miRNA signatures might be useful as diagnostic tool as well as could serve as therapeutic target in HPV induced oncogenesis. Furthermore, distinct characteristics of miRNAs such as resistant to temperature, degradation and non-immunogenic nature to utilize host regulatory machinery make them more appropriate to serve as prognostic indicator of cancer aggressiveness.

Some approaches like restoring miRNA level or blocking its function can be used in drugs development. Concluding same, certain miRNA based cancer-targeted drugs such as miR-34-mimic (MRX34); a liposome-based drug is in clinical trial for the treatment of hepatocellular carcinoma. Likewise, “miravirsen”, antisense oligonucleotides based an antiviral compound known as locked nucleic acids (LNA anti-miRs) is in clinical evaluation for the treatment of hepatitis c virus (HCV)^43^,^44^. Here, we are providing set of HPV regulated miRNAs from various carcinomas and their expression profile, which could be useful in further research to develop novel cancer screening strategies and therapeutic measures.

## Discussion

**Figure 5:**
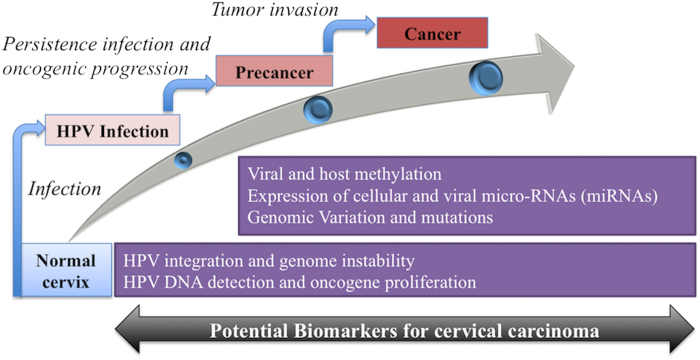
Multistage neoplastic progression and biomarkers overview.

HPVs play a cardinal role in the etiology of distinct carcinomas including CC, HNSCC etc. CC is still account as fourth most common cancer globally and remained as leading cause of cancer mortalities. However, regardless of substantial efforts in the field to develop screening and prevention methods, there is no effectual solution is available to combat against these lethal diseases. The general progression of HPV mediated cancer is associated with the various events and changes with other risk factors. These events can be used as biomarkers. Here, the main aim and feature of HPVbase is to deliver integrative and multi comparative platform for HPV integrations, methylation patterns and cellular miRNAs expression to facilitate identification and analysis of valuable cancer biomarkers. These are very essential factors especially in cervical progression (see Fig. 5).

The HPVbase is the first web knowledgebase to provide highly interactive and manually curated resource for the clinically significant viral and cellular biomarkers. Nevertheless, we have also searched and compared existing sources related to these events and papillomaviruses. For viral integration sites, DR. VIS^45^,^46^ is the only database reported till now, which also host HPV associated entries. However, in HPVbase, we have compiled 1257 integration and breakpoint events associated with different clinical and histological conditions. Whereas, we found 8 methylation related databases (see additional file 1, Table S1). All these databases only address host specific methylation patterns. However, we are first time providing integrated resource for the HPV methylome profile in different carcinomas with relevant clinical information. Simultaneously, there are mainly 19 data sources is available related to microRNAs (see additional file 1, Table S2), from which no one provides information pertinent to HPV mediated aberrant expression of cellular microRNAs in distinct carcinomas. Here, we are presenting a comprehensive catalogue of 341 deregulated miRNAs with additional knowledge such as histological condition, potential target genes; their chromosomal locations etc. This can be valuable for the development of miRNA-based therapeutics.

Some sources also developed which provide HPV genomic and proteomic information. The Papillomavirus Episteme (PaVE) contains 303 genomes, 3126 gene region, 2823 protein sequences and 48 protein structures (http://pave.niaid.nih.gov)^47^. The Human papillomavirus T cell Antigen Database (HPVdb) hosts 2781 antigen entries of antigenic proteins, 191 T cell epitopes and 45 human leukocytes antigen (HLA) ligands with alignment and search tools (http://cvc.dfci.harvard.edu/hpv/)^48^. However, no resource is available related to HPV mediated cancer biomarkers. To support cancer biomarker discovery and utilize existing knowledge and information, we have developed a web-based interactive resource “***HPVbase”*** for the extensive comparative analysis of different biomarkers and genomic information. It will provide systematic compilation of knowledge and user-friendly visualization of clinical content. It can be exploited for assessment of clinically significant biomarker data and will provide a comprehensive base for examination between distinct features and cancers, which in turn facilitate advancement in clinical screening tests. Finally, combined impact of multiple events can be utilized for the future prevention and therapeutics. Thus, in turn this exhaustive platform promises to be precious to facilitate and accelerate reliable cancer diagnostics and prognosis.

## Materials and methods

### Biomarker data collection and curation

A systematic and extensive search was performed using keywords from PubMed. All exhaustive clinical studies reporting (1) the integration and breakpoint events in HPV associated carcinomas (2) HPVs methylation events relevant to the cancer clinical outcomes and (3) the HPV mediated aberrant expression of distinct host miRNAs that are crucial in cancer etiology were included and analyzed.

### HPV integration and breakpoints

Collection of integration and breakpoints were derived from scientific literatures searched using set of keywords “((((((HPVs) OR human papillomaviruses) OR human papillomavirus) OR HPV*)) AND (((cancer) OR carcinoma))) AND ((integration*) OR breakpoint*)” as query. Overall, 755 articles were found, out of which 117 review articles were excluded from study and 638 research papers were screened for extensive data mining. Furthermore, maximum studies only state HPV status or the information related to presence or absence of HPV genes such studies does not provide integration sites, coordinates and further clinical details were filtered and removed. After vigilant reading, comprehensive knowledge associated with integration events were extracted such as HPV type (e.g., HPV16, HPV18), HPV region (e.g., E6), viral integrant/breakpoints (e.g., 84–107), human chromosome number (e.g., 3) and coordinates (e.g., 47984368–47984397), cytoband (e.g., q21.3), target genes (e.g., SYN3, PROX1), fragile sites (e.g., FRA2K), detection technique or methods (e.g., RNA-seq, APOT assay), histological conditions (e.g., CC, HNSCC), specimen or sample type (e.g., tumor sample, cell line) etc. The collected data were manually checked and rectified for errors and inconsistencies.

### HPV DNA methylation patterns

The HPV DNA methylation studies were extensively searched using combination of keywords “(((((((HPVs) OR human papillomaviruses) OR human papillomavirus) OR HPV*))) AND (((cancer) OR carcinoma))) AND methylation”. In total, 320 articles excluding 31 review articles were screened. Most of the literature covers host genome or gene methylations, which are not in the scope of this study and hence excluded. All-inclusive information related to HPV methylation i.e. HPV type (e.g., HPV16), its genomic region (e.g., E2), methylation status (e.g., HyperM), technique used for detecting methylation (e.g., bisulphite sequencing), sample type (e.g., clinical biopsy samples), associated cancer type (e.g., CC) or stage (e.g., cervical intraepithelial neoplasia (CIN)) etc. were mined and compiled.

### Cellular miRNAs aberrant expression

All literature information were collected using keywords query “((((((HPVs) OR human papillomaviruses) OR human papillomavirus) OR HPV*)) AND ((cancer) OR carcinoma)) AND ((((microRNA) OR miRNA) OR microRNAs) OR miRNAs)” from PubMed. Overall, 99 articles excluding 24 review articles were searched. We have extracted information comprises of miRNAs, their expression profile, mirbase id, host chromosomal location and genome coordinates from distinct human carcinomas. Apart from manually curated expression profiles, these are further integrated and linked with the external resources. Along with this, we also explored and obtained known target genes of different miRNAs using mirtarbase^49^.

### HPVbase interface and construction

The web interface (front and back end) is developed using the open source LAMP (Linux-Apache-MySQL-PHP) solution stack reinforced by combination of programming and scripting languages i.e. HTML, javascript and PERL. We have also utilized the JBrowse^50^, a java-script based genome browser to develop descriptive sections related to specific biomarkers using highly lightweight interchangeable JavaScript Object Notation (JSON) data format. The all data files were converted using custom Perl script in gene feature files (GFF3) format with some manual editing and configured using JBrowse to visualize interactively. The complete system hosted on IBM machine under Red Hat Enterprise Linux 5 environment using MySQL client version 5.0.51 b, PHP 5.2.14 and Apache 2.2.17 server.

### HPVbase organization and search tools

**Figure 6:**
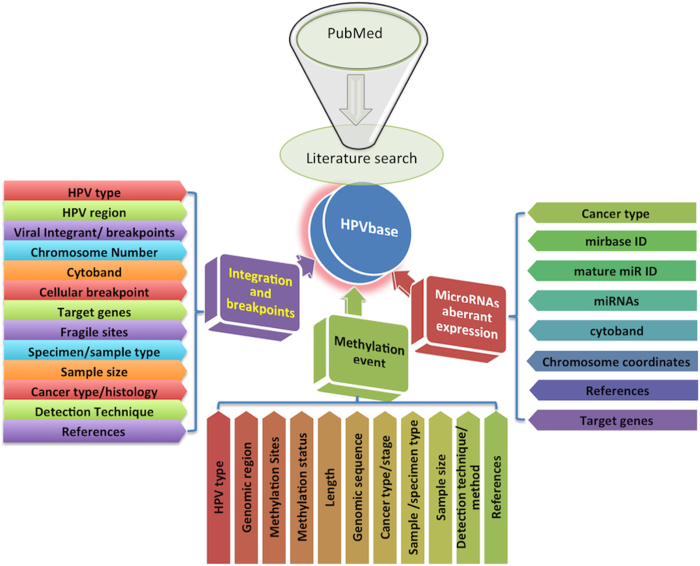
Layout depicting overall organization of HPVbase.

HPVbase is a first web-based, HPV knowledgebase for the effective analysis and assessment of viral and cellular biomarkers involves in pathogenicity of different HPV mediated carcinomas. It has well-designed user interface for the interactive visualization and evaluation. In HPVbase, data is classified and organized in three different sections- integration sites and breakpoints, methylation patterns and miRNAs expression profiles (see Fig. 6). Subsequently, these biomarkers are further divided into various subsections based on the viral types and diverse carcinomas. Each biomarker sections are represented in highly interactive browsers and user intensive tabular interfaces with other useful external links. It provides user friendly searching and browsing facility for data retrieval. Each section encompasses search box and sorting ability. User can search and filter data using any relevant keywords like specific HPV type, HPV genomic region, cytoband, target genes, detection technique used etc. (e.g., HPV18, E7, q32.3, RNA-seq). It also facilitates browsing of clinically important data based on the HPV genotypes and individual carcinoma.

Alongside, HPVbase also facilitate user affable data searching and retrieval options. The resulting two search tools (a) integration search and (b) advance search have been implemented at website.

#### (a) Integration search

User can extensively search integration sites based on various keywords with exact or containing sub-options from number of provided fields including integration site, HPV type, chromosomal locations, cancer type etc.

#### (b) Advance search

This tool permits user to perform systematic search using combination of logical operators (OR/AND). It allows user to build a query using different combination of keywords to narrow down search.

### Updating of HPVbase

We have incorporated the latest and highly curated data in HPVbase. To cope with this increasing amount of valuable clinical data, we would like to further update our resource in every six-month.

## Additional Information

**How to cite this article**: Kumar Gupta, A. and Kumar, M. HPVbase- a knowledgebase of viral integrations, methylation patterns and microRNAs aberrant expression: As potential biomarkers for Human papillomaviruses mediated carcinomas. *Sci. Rep.* **5**, 12522; doi: 10.1038/srep12522 (2015).

## Supplementary Material

Supplementary Information
