# Supplementary material for: HPVbase – a knowledgebase of viral integrations, methylation patterns and microRNAs aberrant expression: As potential biomarkers for Human papillomaviruses mediated carcinomas
Source: Sci Rep. 2015 Jul 24;5:12522. doi: 10.1038/srep12522 (PMC4513345; doi:10.1038/srep12522)
Supplement: Supplementary Information [file srep12522-s1.pdf]

HPVbase – A knowledgebase of viral integrations, methylation patterns and microRNAs aberrant expression: As potential biomarkers in Human papillomaviruses mediated carcinomas

Amit Kumar Gupta[1] and Manoj Kumar[1,*]

[1]Bioinformatics Centre, Institute of Microbial Technology, Council of Scientific and Industrial Research (CSIR), Sector 39-A, Chandigarh-160036, India

* To whom correspondence should be addressed. Tel, 91-172-6665453; Fax, 91-172-2690585; 91-172-2690632; Email, manojk@imtech.res.in

Additional files

Additional file 1

Figure S1 Screenshot showing integration site mapped on the HPV16 (NC_001526.2) reference genome, along with detailed description such as human genome position, region of HPV genome, junction sequence, source, integrated HPV DNA sequence and length.

Figure S2 Distribution of integration sites among distinct HPV types.

Figure S3 Distribution of integration sites among distinct cancer types.

Figure S4 Representing number of integration sites distributed at major genomic loci regions.

Figure S5 A screenshot depicting integration data with corresponding clinical annotations and reference information in tabular format.

Figure S6 Screenshot showing highly interactive and user intensive methylation browser with associated histological information.

Figure S7 Graph showing distribution of methylation sites among distinct HPV types.

Figure S8 An example screenshot illustrating HPV mediated upregulated miRNAs expression profile and analysis with interconnected external links.

Table S1 List of available major resources specific for methylation data.

Table S2 List of available major resources related to microRNA data.

Table S3 List of human genes altered or disrupted due to HPV integration events.

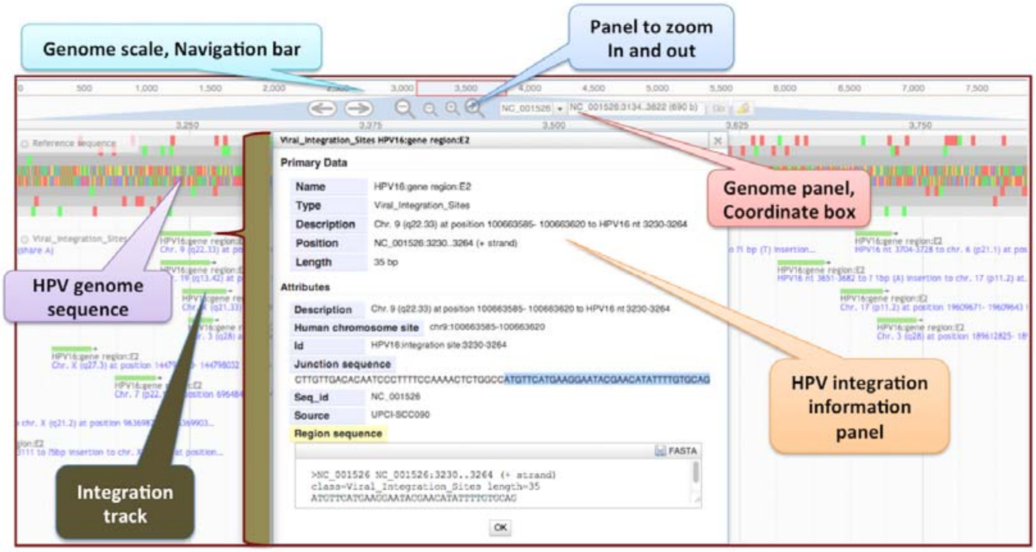

Figure S1 Screenshot showing integration site mapped on the HPV16 (NC_001526.2) reference genome, along with detailed description such as human genome position, region of HPV genome, junction sequence, source, integrated HPV DNA sequence and length.

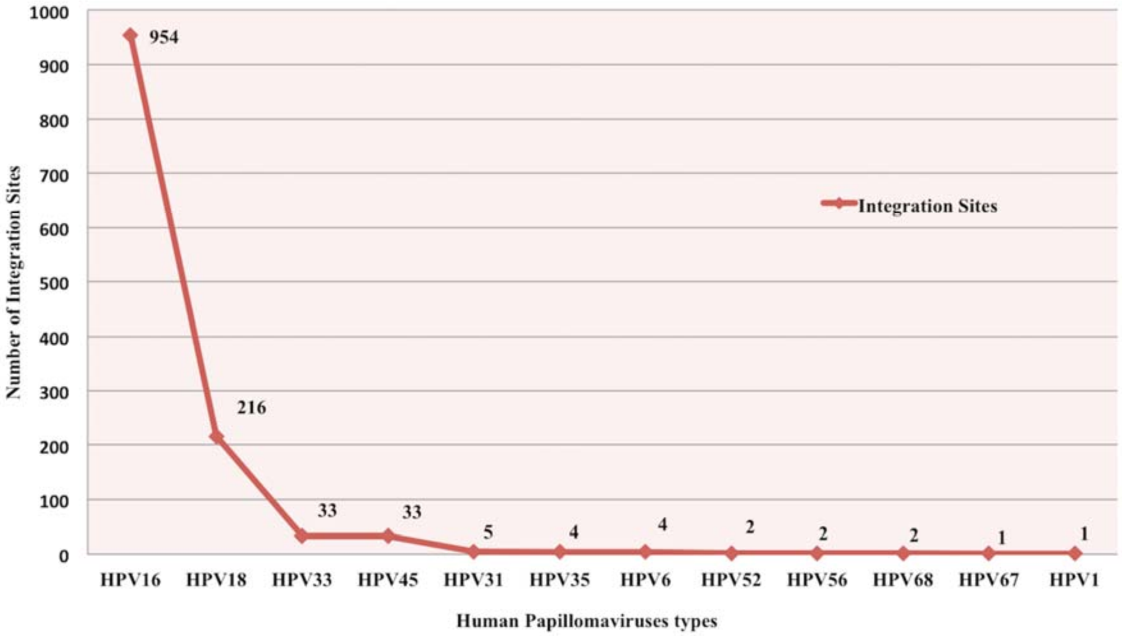

Figure S2 Distribution of integration sites among distinct HPV types.

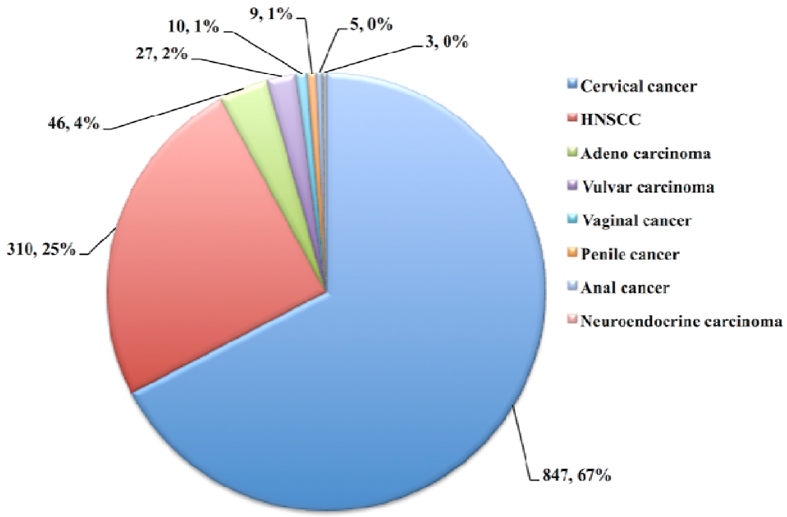

Figure S3 Distribution of integration sites among distinct cancer types.

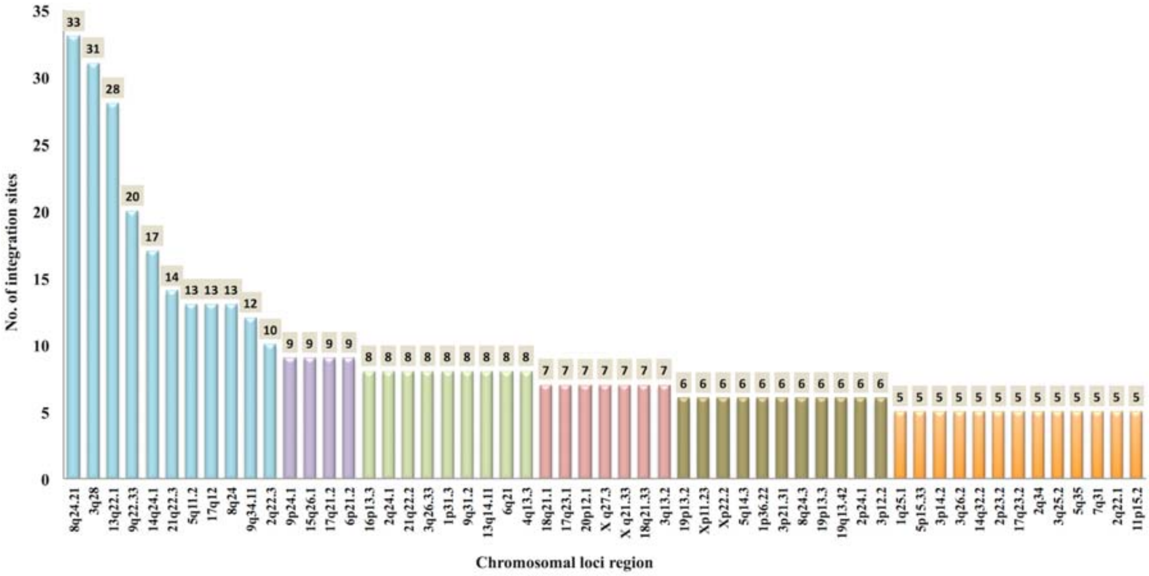

Figure S4 Representing number of integration sites distributed at major genomic loci regions.

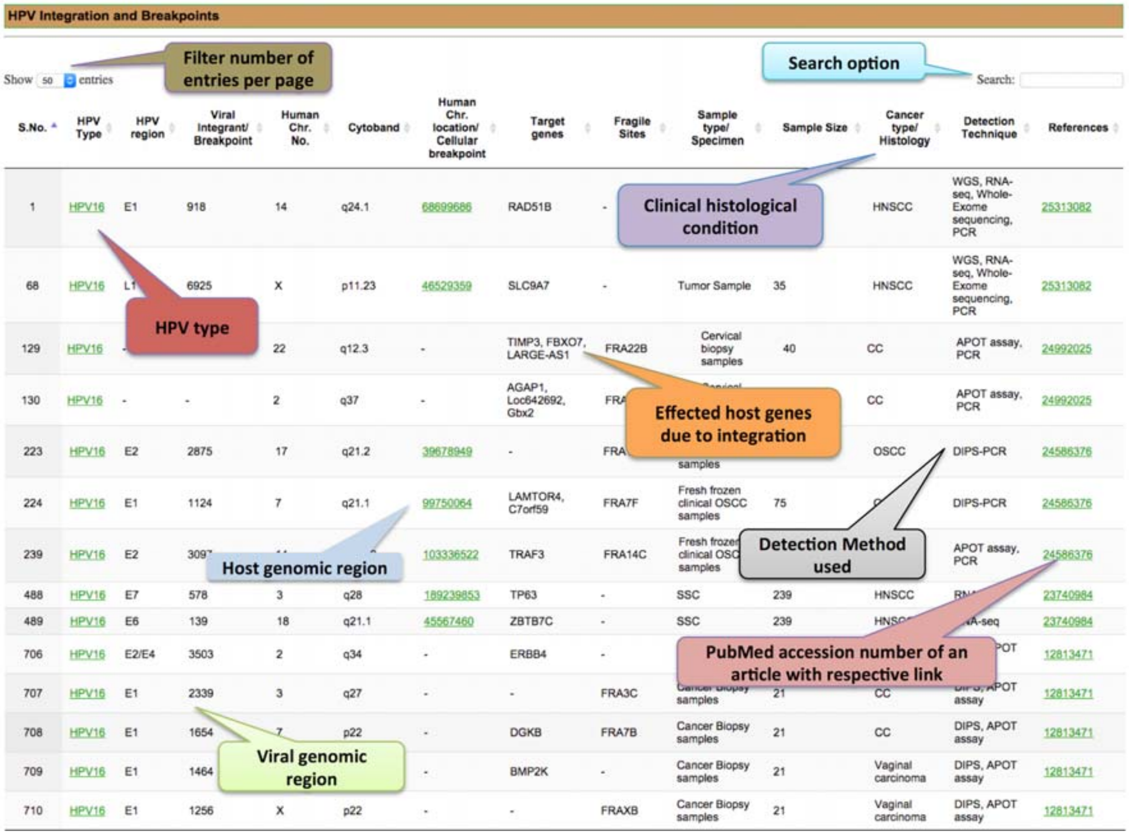

Figure S5 A screenshot depicting integration data with corresponding clinical annotations and reference information in tabular format.

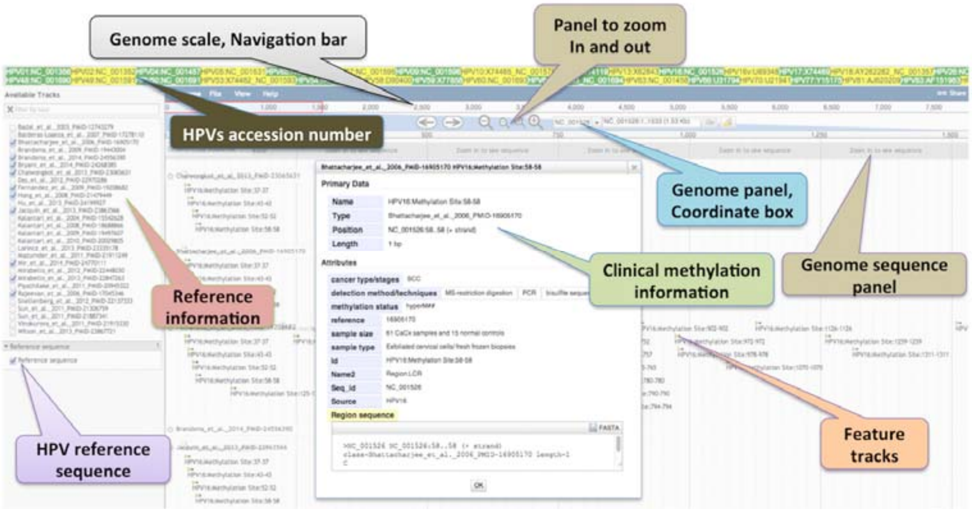

Figure S6 Screenshot showing highly interactive and user intensive methylation browser with associated histological information.

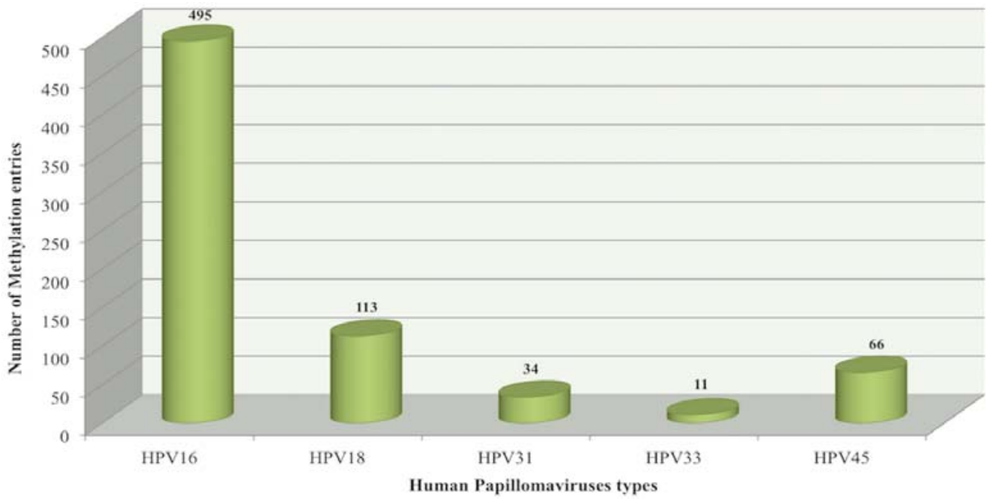

Figure S7 Graph showing distribution of methylation sites among distinct HPV types.

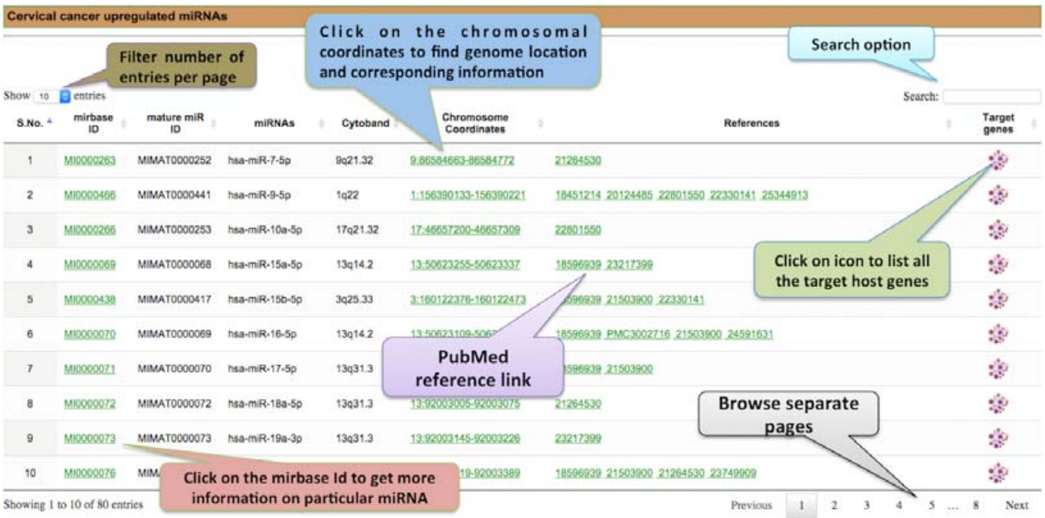

Figure S8 An example screenshot illustrating HPV mediated upregulated miRNAs expression profile and analysis with interconnected external links.

Table S1 List of available major resources specific for methylation data.

Database Name	References
MethDB	[1]
MethyCancer	[2]
MethCancerDB	[3]
PubMeth	[4]
DiseaseMeth	[5]
MethylomeDB	[6]
NGSmethDB	[7]
PCMdb	[8]

Table S2 List of available major resources related to microRNA data.

Database Name	References
miRGen	[9]
ViTa	[10]
miRNAMap	[11]
MicroRNA.org	[12]
Vir-Mir db	[13]
miRecords	[14]
miROrtho	[15]
miR2Disease	[16]
PMRD	[17]
TransmiR	[18]
PmiRKB	[19]
mESAdb	[20]
TarBase	[21]
miREX	[22]
miRGator	[23]
mirbase	[24]
miRTarBase	[25]
miRNEST	[26]
HMDD v2.0	[27]

References:

1 Amoreira, C., Hindermann, W. & Grunau, C. An improved version of the DNA Methylation database (MethDB). *Nucleic Acids Res* **31**, 75-77 (2003).

2 He, X. *et al.* MethyCancer: the database of human DNA methylation and cancer. *Nucleic Acids Res* **36**, D836-841; DOI:10.1093/nar/gkm730 (2008).

3 Lauss, M. *et al.* MethCancerDB--aberrant DNA methylation in human cancer. *Br J Cancer* **98**, 816-817; DOI:10.1038/sj.bjc.6604219 (2008).

4 Ongenaert, M. *et al.* PubMeth: a cancer methylation database combining text-mining and expert annotation. *Nucleic Acids Res* **36**, D842-846; DOI:10.1093/nar/gkm788 (2008).

5 Lv, J. *et al.* DiseaseMeth: a human disease methylation database. *Nucleic Acids Res* **40**, D1030-1035; DOI:10.1093/nar/gkr1169 (2012).

6 Xin, Y. *et al.* MethylomeDB: a database of DNA methylation profiles of the brain. *Nucleic Acids Res* **40**, D1245-1249; DOI:10.1093/nar/gkr1193 (2012).

7 Geisen, S., Barturen, G., Alganza, A. M., Hackenberg, M. & Oliver, J. L. NGSmethDB: an updated genome resource for high quality, single-cytosine resolution methylomes. *Nucleic Acids Res* **42**, D53-59; DOI:10.1093/nar/gkt1202 (2014).

8 Nagpal, G. *et al.* PCMdb: pancreatic cancer methylation database. *Sci Rep* **4**, 4197; DOI:10.1038/srep04197 (2014).

9 Megraw, M., Sethupathy, P., Corda, B. & Hatzigeorgiou, A. G. miRGen: a database for the study of animal microRNA genomic organization and function. *Nucleic Acids Res* **35**, D149-155; DOI:10.1093/nar/gkl904 (2007).

10 Hsu, P. W., Lin, L. Z., Hsu, S. D., Hsu, J. B. & Huang, H. D. ViTa: prediction of host microRNAs targets on viruses. *Nucleic Acids Res* **35**, D381-385; DOI:10.1093/nar/gkl1009 (2007).

11 Hsu, S. D. *et al.* miRNAMap 2.0: genomic maps of microRNAs in metazoan genomes. *Nucleic Acids Res* **36**, D165-169; DOI:10.1093/nar/gkm1012 (2008).

12 Betel, D., Wilson, M., Gabow, A., Marks, D. S. & Sander, C. The microRNA.org resource: targets and expression. *Nucleic Acids Res* **36**, D149-153; DOI:10.1093/nar/gkm995 (2008).

13 Li, S. C., Shiau, C. K. & Lin, W. C. Vir-Mir db: prediction of viral microRNA candidate hairpins. *Nucleic Acids Res* **36**, D184-189; DOI:10.1093/nar/gkm610 (2008).

14 Xiao, F. *et al.* miRecords: an integrated resource for microRNA-target interactions. *Nucleic Acids Res* **37**, D105-110; DOI:10.1093/nar/gkn851 (2009).

15 Gerlach, D., Kriventseva, E. V., Rahman, N., Vejnar, C. E. & Zdobnov, E. M. miROrtho: computational survey of microRNA genes. *Nucleic Acids Res* **37**, D111-117; DOI:10.1093/nar/gkn707 (2009).

16 Jiang, Q. *et al.* miR2Disease: a manually curated database for microRNA deregulation in human disease. *Nucleic Acids Res* **37**, D98-104; DOI:10.1093/nar/gkn714 (2009).

17 Zhang, Z. *et al.* PMRD: plant microRNA database. *Nucleic Acids Res* **38**, D806-813; DOI:10.1093/nar/gkp818 (2010).

18 Wang, J., Lu, M., Qiu, C. & Cui, Q. TransmiR: a transcription factor-microRNA regulation database. *Nucleic Acids Res* **38**, D119-122; DOI:10.1093/nar/gkp803 (2010).

19 Meng, Y. *et al.* PmiRKB: a plant microRNA knowledge base. *Nucleic Acids Res* **39**, D181-187; DOI:10.1093/nar/gkq721 (2011).

20 Kaya, K. D., Karakulah, G., Yakicier, C. M., Acar, A. C. & Konu, O. mESAdb: microRNA expression and sequence analysis database. *Nucleic Acids Res* **39**, D170-180; DOI:10.1093/nar/gkq1256 (2011).
21 Vergoulis, T. *et al.* TarBase 6.0: capturing the exponential growth of miRNA targets with experimental support. *Nucleic Acids Res* **40**, D222-229; DOI:10.1093/nar/gkr1161 (2012).
22 Bielewicz, D. *et al.* mirEX: a platform for comparative exploration of plant pri-miRNA expression data. *Nucleic Acids Res* **40**, D191-197; DOI:10.1093/nar/gkr878 (2012).
23 Cho, S. *et al.* MiRGator v3.0: a microRNA portal for deep sequencing, expression profiling and mRNA targeting. *Nucleic Acids Res* **41**, D252-257; DOI:10.1093/nar/gks1168 (2013).
24 Kozomara, A. & Griffiths-Jones, S. miRBase: annotating high confidence microRNAs using deep sequencing data. *Nucleic Acids Res* **42**, D68-73; DOI:10.1093/nar/gkt1181 (2014).
25 Hsu, S. D. *et al.* miRTarBase update 2014: an information resource for experimentally validated miRNA-target interactions. *Nucleic Acids Res* **42**, D78-85; DOI:10.1093/nar/gkt1266 (2014).
26 Szczesniak, M. W. & Makalowska, I. miRNEST 2.0: a database of plant and animal microRNAs. *Nucleic Acids Res* **42**, D74-77; DOI:10.1093/nar/gkt1156 (2014).
27 Li, Y. *et al.* HMDD v2.0: a database for experimentally supported human microRNA and disease associations. *Nucleic Acids Res* **42**, D1070-1074; DOI:10.1093/nar/gkt1023 (2014).

Table S3 List of human genes altered or disrupted due to HPV integration events.

S. No.	Gene Name	Frequency
1	MYC	15
2	TP63	13
3	RAD51B	12
4	KLF12	10
5	KLF5	9
6	ETS2	7
7	TCTEX1D1	7
8	FHIT	7
9	DIAPH2	7
10	HEMGN	7
11	ZBTB7C	6
12	ACTL7B	6
13	IFT140	5
14	CRAT	5
15	SLC9A7	5
16	CEACAM5	5
17	BRSK1	5
18	SERPINB3	5
19	LRP1B	5
20	EPSTI1	5
21	TEAD1	5
22	PHLDB2	5
23	CD274	4
24	FANCC	4
25	ERBB2	4
26	LEPREL1	4
27	TUBD1	4
28	CASZ1	4
29	POU5F1B	4
30	ANPEP	4
31	PLCXD2	4
32	ASIC2	4
33	NR4A2	3
34	FRMPD4	3
35	MRPS31	3
36	BCL11B	3

S. No.	Gene Name	Frequency
241	PKP2	1
242	MEX3B	1
243	B4GALNT2	1
244	TLE4	1
245	TMPRSS11B	1
246	STK11	1
247	MACC1	1
248	KCND3	1
249	SLC6A14	1
250	IL1RAPL1	1
251	Mar-10	1
252	PAEP	1
253	FBXL7	1
254	TBX22	1
255	SNX19	1
256	DPF3	1
257	ABCA13	1
258	ZXDA	1
259	SLC26A8	1
260	P2RY1	1
261	ABCB10	1
262	HNRPDL	1
263	PDE7B	1
264	MAP3K5	1
265	TYW1	1
266	SLC25A25	1
267	PTEN	1
268	POMP	1
269	HNF1B	1
270	GOLPH3L	1
271	NID1	1
272	TP73	1
273	CDH10	1
274	PKHD1	1
275	MPPED2	1
276	LRRC28	1

37	RARA	3
38	SLITRK2	3
39	VMP1	3
40	MACROD2	3
41	GPN1	3
42	IL8	3
43	ERBB4	3
44	EYA2	3
45	COX4I2	3
46	MYCN	3
47	RREB1	3
48	GRB7	3
49	LYST	3
50	FOXO1	3
51	ACCN1	3
52	PRKRIR	2
53	SERPINB4	2
54	IQGAP1	2
55	MAGI2	2
56	TRPC4AP	2
57	PRDX5	2
58	EBAG9	2
59	COX6B1	2
60	UPK1A	2
61	DAPK1	2
62	PROX1	2
63	TNIK	2
64	AGO2	2
65	STARD3	2
66	BRE	2
67	JUP	2
68	INO80C	2
69	ATXN2	2
70	SEMA4B	2
71	UBAP2	2
72	GATAD2A	2
73	USP4	2
74	LIPC	2
75	ORC2	2

277	THSD4	1
278	CCR7	1
279	BRCA1	1
280	PHF12	1
281	ATP8B1	1
282	SLC9A8	1
283	LPP	1
284	DUX4	1
285	INPP4B	1
286	MAPK10	1
287	JAK2	1
288	CDIP1	1
289	RFFL	1
290	MALT1	1
291	BSPH1	1
292	SEZ6L	1
293	FAM110B	1
294	KIAA1432	1
295	EIF1	1
296	ZNF506	1
297	CEACAM6	1
298	CAMK1G	1
299	UMOD	1
300	PIM1	1
301	CDH13	1
302	ZEB2	1
303	MAP4	1
304	DGKB	1
305	BMP2K	1
306	RANBP9	1
307	ZDHHC21	1
308	SI	1
309	MUC5B	1
310	KCNIP1	1
311	MYF5	1
312	NOTCH1	1
313	RIPK2	1
314	ELK3	1
315	ODC1	1

76	MECOM	2
77	RPA3	2
78	MIOS	2
79	SLC47A2	2
80	PDE4D	2
81	CTIF	2
82	OR1M1	2
83	TNFSF4	2
84	ATP11A	2
85	SLC25A36	2
86	SOX2	2
87	DRAP1	2
88	GMPS	2
89	SRGAP1	2
90	Nf1	2
91	WASF2	2
92	GLS	2
93	XPR1	2
94	SLC22A23	2
95	EHF	2
96	SERPINB2	2
97	JAG1	2
98	TMCC3	2
99	KLHL28	2
100	ITGB6	2
101	SLC1A3	2
102	EPHX2	2
103	PRKACA	2
104	CD34	2
105	FAM120B	2
106	LRFN5	2
107	ESRRA	2
108	PKHD1L1	2
109	ANAPC16	2
110	EIF2C2	2
111	MDM2	2
112	MYOF	2
113	FAM179B	2
114	CCDC88B	2

316	RPS27	1
317	PTPN13	1
318	KLHL3	1
319	TCP1	1
320	KPNA3	1
321	AFP	1
322	MED24	1
323	TERT	1
324	TTLL7	1
325	TRAP1	1
326	FAM92A1	1
327	PTPRD	1
328	ZSCAN5A	1
329	CDKAL1	1
330	MSI2	1
331	USP21	1
332	ZFAND3	1
333	ALS2CR11	1
334	MYO1D	1
335	TNFAIP2	1
336	ARCN1	1
337	SH3PXD2A	1
338	RHEB	1
339	GPAA1	1
340	FOXK1	1
341	SNIP1	1
342	FMO3	1
343	RAP2B	1
344	MAFA	1
345	EGFL7	1
346	KRT39	1
347	TBL1XR1	1
348	CYS1	1
349	KCNIP4	1
350	SLC7A11	1
351	ARHGAP26	1
352	SH3TC2	1
353	IMMP2L	1
354	NPM3	1

115	SYBU	2	355	SSPN	1
116	CDK4	2	356	HAUS4	1
117	SERPINB7	1	357	AGBL1	1
118	DOLPP1	1	358	FLRT3	1
119	PLGRKT	1	359	SMARCA1	1
120	ARHGDIA	1	360	DYX1C1	1
121	CCDC39	1	361	NFIX	1
122	TTC14	1	362	ZNF277	1
123	CEACAM19	1	363	LRSAM1	1
124	GRIK1	1	364	PCDH7	1
125	KIAA1279	1	365	DEPTOR	1
126	ARHGEF10	1	366	HEATR1	1
127	ANGPT1	1	367	RNGTT	1
128	SLC41A2	1	368	NUP35	1
129	RFX7	1	369	SH3BP5	1
130	THSD1	1	370	PALB2	1
131	EXOC7	1	371	TMEM114	1
132	ROCK2	1	372	BCL2L13	1
133	MSH2	1	373	COG8	1
134	COG4	1	374	MRVI1	1
135	WWOX	1	375	MID1	1
136	ADAMTS18	1	376	DNAJC15	1
137	MAP1LC3B	1	377	PTHLH	1
138	LMBR1	1	378	KLF7	1
139	TNC	1	379	GPD2	1
140	ASCC1	1	380	CXCL6	1
141	ZNF365	1	381	CFTR	1
142	SETBP1	1	382	PCDH9	1
143	MAK	1	383	AKR1C3	1
144	ACTN4	1	384	MPZL3	1
145	TLL1	1	385	DDX21	1
146	Egr1	1	386	ETV2	1
147	FANCM	1	387	VPS36	1
148	PIBF1	1	388	FOXJ1	1
149	TIMP3	1	389	EPCAM	1
150	AGAP1	1	390	SF3B3	1
151	CD28	1	391	NUDT7	1
152	EIF2AK2	1	392	FBXO31	1
153	CACNA1E	1	393	NOM1	1

154	DENND6A	1	394	RP9	1
155	SEMA3E	1	395	Dec-01	1
156	RHOH	1	396	RTKN2	1
157	INSR	1	397	GCM2	1
158	CCNE1	1	398	EIF3K	1
159	GLI2	1	399	Reep2	1
160	ARID1B	1	400	BORA	1
161	BLVRA	1	401	FBXO7	1
162	SNX30	1	402	SULT6B1	1
163	POC1B	1	403	SEMA3A	1
164	DACH1	1	404	N4BP2	1
165	PARN	1	405	PLEKHF1	1
166	P4HB	1	406	COA1	1
167	PTPRT	1	407	BFAR	1
168	USP9X	1	408	TCAP	1
169	GATA5	1	409	CDH9	1
170	GOLT1A	1	410	CCDC50	1
171	ERC2	1	411	FBXW7	1
172	EPHA7	1	412	NKX6-3	1
173	SYN3	1	413	TSC2	1
174	HCN4	1	414	BCL3	1
175	HDAC2	1	415	CHEK2	1
176	SGSM1	1	416	RPS6KB1	1
177	GARS	1	417	BCKDHB	1
178	LAMTOR4	1	418	SCN2B	1
179	LHFPL3	1	419	KBTBD11	1
180	PNRC1	1	420	ZBTB32	1
181	DEPDC5	1	421	CHST11	1
182	TRAF3	1	422	GPR157	1
183	PHF21B	1	423	ZACN	1
184	DUX4L2	1	424	PQLC3	1
185	DENND1A	1	425	FUK	1
186	DCAF12L2	1	426	MAF	1
187	ATP11B	1	427	RNF32	1
188	PDK3	1	428	FKBP9	1
189	BCL2L14	1	429	TNFSF8	1
190	USP6NL	1	430	SPOCK2	1
191	ARID3A	1	431	ADO	1
192	CLDN1	1	432	CAPN12	1

193	CENPW	1
194	LOXL4	1
195	SLC29A1	1
196	PARD3B	1
197	HIF1A	1
198	ULK2	1
199	IFT74	1
200	PYROXD2	1
201	MRPL1	1
202	CSMD3	1
203	MBD5	1
204	STS	1
205	NHS	1
206	CREB5	1
207	FHOD3	1
208	PID1	1
209	RUNX2	1
210	PTPRJ	1
211	CBFA2T2	1
212	MOB3A	1
213	URI1	1
214	MDGA2	1
215	KHK	1
216	STXBP6	1
217	FAM3C	1
218	EDEM1	1
219	MYOC	1
220	P2RY2	1
221	KARS	1
222	NPTXR	1
223	HMGCR	1
224	MUC4	1
225	FIG4	1
226	RGS10	1
227	SRRM4	1
228	PEBP4	1
229	SCFD1	1
230	ARHGEF4	1
231	NOL10	1

433	SPOCK3	1
434	PCDHB1	1
435	Gbx2	1
436	HEATR5B	1
437	ZNF648	1
438	ARF4	1
439	PCLO	1
440	CHRNA9	1
441	MBD3L3	1
442	PLA2G10	1
443	PNMT	1
444	ANK1	1
445	CIITA	1
446	ERCC2	1
447	EWSR1	1
448	PGAP3	1
449	IKZF3	1
450	AKT2	1
451	TPRG1	1
452	TYRP1	1
453	NRXN1	1
454	BZW1	1
455	MDS1	1
456	CLDN16	1
457	NANOS3	1
458	ARX	1
459	BMP8	1
460	MAP2	1
461	DYSF	1
462	RTN4IP1	1
463	TCRA	1
464	LEU2	1
465	PCDH8	1
466	ZNF174	1
467	CKAP2	1
468	PGF	1
469	LTBP2	1
470	NUMB	1
471	RGNEF	1

232	WHSC1	1
233	DEK	1
234	RFWD2	1
235	SGIP1	1
236	SLC24A4	1
237	COL23A1	1
238	HRH1	1
239	KDM6B	1
240	PDZD8	1

472	UBN2	1
473	FXR2	1
474	TMEM49	1
475	CNN2	1
476	HOX11	1
477	LYT10	1
478	PAX2	1
479	APM1	1
480	DCC	1
481	DPC4	1
